# About Microangiopathic Hemolytic Anemia

**DOI:** 10.4274/tjh.2014.0382

**Published:** 2015-02-15

**Authors:** Şinasi Özsoylu

**Affiliations:** 1 Retired Professor of Pediatrics, Hematology, and Hepatology, Honorary Fellow of American Academy of Pediatrics, Honorary Member of American Pediatric Society

**Keywords:** Microangiopathy, hemolytic anemia

## TO THE EDITOR

I have read with interest the extensive review article by Yenerel on atypical hemolytic uremic syndrome (aHUS) in a recent issue of this journal [1].

I would like to bring to attention the point that more recently some authors do not use the term ‘aHUS’, which was historically used to distinguish heterogeneous, uncharacterized syndromes from Shiga toxin-related HUS (ST-HUS), since the term lacks both specificity and suggested causes [2].

I would also like to emphasize the term ‘hereditary’ instead of ‘congenital’ thrombotic thrombocytopenic purpura (TTP). I believe that ‘hereditary’ is more appropriate since hereditary factors are involved in these conditions. ‘Congenital’ seems to be more appropriate for conditions without gene involvement such as congenital syphilis, congenital tuberculosis, congenital rubella, etc., as I have brought to attention on several earlier occasions [3,4].

In the pathogenesis of thrombotic microangiopathic hemolytic anemia (TMHA) syndromes, endothelial injury and complement regulation (fluid phase and membrane attack) are causes of attacks that should not be omitted.

Although eculizumab was emphasized, which is currently available as an anticomplementary agent, it is extremely expensive and may be limited among patients with C5 mutations [2,5].

It should be noted that glucocorticoids are standard treatment for TMHA. We have also used mega-dose methylprednisolone successfully in a patient on 3 different occasions. Rituximab and other immunosuppressive agents are appropriate when the clinical course is complicated.
